# Multimodal Imaging Detection of Difficult Mammary Paget Disease: Dermoscopy, Reflectance Confocal Microscopy, and Line-Field Confocal–Optical Coherence Tomography

**DOI:** 10.3390/diagnostics15151898

**Published:** 2025-07-29

**Authors:** Carmen Cantisani, Gianluca Caruso, Alberto Taliano, Caterina Longo, Giuseppe Rizzuto, Vito D’Andrea, Pawel Pietkiewicz, Giulio Bortone, Luca Gargano, Mariano Suppa, Giovanni Pellacani

**Affiliations:** 1 UOC of Dermatology, Policlinico Umberto I Hospital, Sapienza Medical School of Rome, 00161 Roma, Italy; albertotaliano1@gmail.com (A.T.); giuseppe.rizzuto@uniroma1.it (G.R.); giuliobortone93@gmail.com (G.B.); lucagargano1995@gmail.com (L.G.); giovanni.pellacani@uniroma1.it (G.P.); 2 Unit of Cellular Pathology, San Filippo Neri Hospital of Rome, 00135 Roma, Italy; gianluca.caruso79@gmail.com; 3 Department of Dermatology, University of Modena and Reggio Emilia, 41121 Modena, Italy; caterina.longo@unimore.it; 4 Azienda Unità Sanitaria Locale—IRCCS di Reggio Emilia, Skin Cancer Center, 42122 Reggio Emilia, Italy; 5 General Surgery Department of Surgery, Sapienza University of Rome, 00185 Roma, Italy; vito.dandrea@uniroma1.it; 6 Zwierzyniecka Medical Center, 60-814 Poznań, Poland; pietkiewicz.pp@gmail.com; 7 Department of Dermatology, Hôpital Erasme—Hôpitaux Universitaires de Bruxelles (HUB), 1070 Brussels, Belgium

**Keywords:** dermoscopy, line-field optical-coherence tomography, mammary Paget disease, reflectance confocal microscopy, nipple areola complex

## Abstract

Mammary Paget disease (MPD) is a rare cutaneous malignancy associated with underlying ductal carcinoma in situ (DCIS) or invasive ductal carcinoma (IDC). Clinically, it appears as eczematous changes in the nipple and areola complex (NAC), which may include itching, redness, crusting, and ulceration; these symptoms can sometimes mimic benign dermatologic conditions such as nipple eczema, making early diagnosis challenging. A 56-year-old woman presented with persistent erythema and scaling of the left nipple, which did not respond to conventional dermatologic treatments: a high degree of suspicion prompted further investigation. Reflectance confocal microscopy (RCM) revealed atypical, enlarged epidermal cells with irregular boundaries, while line-field confocal–optical coherence tomography (LC-OCT) demonstrated thickening of the epidermis, hypo-reflective vacuous spaces and abnormally large round cells (Paget cells). These non-invasive imaging findings were consistent with an aggressive case of Paget disease despite the absence of clear mammographic evidence of underlying carcinoma: in fact, several biopsies were needed, and at the end, massive surgery was necessary. Non-invasive imaging techniques, such as dermoscopy, RCM, and LC-OCT, offer a valuable diagnostic tool in detecting Paget disease, especially in early stages and atypical forms.

**Figure 1:**
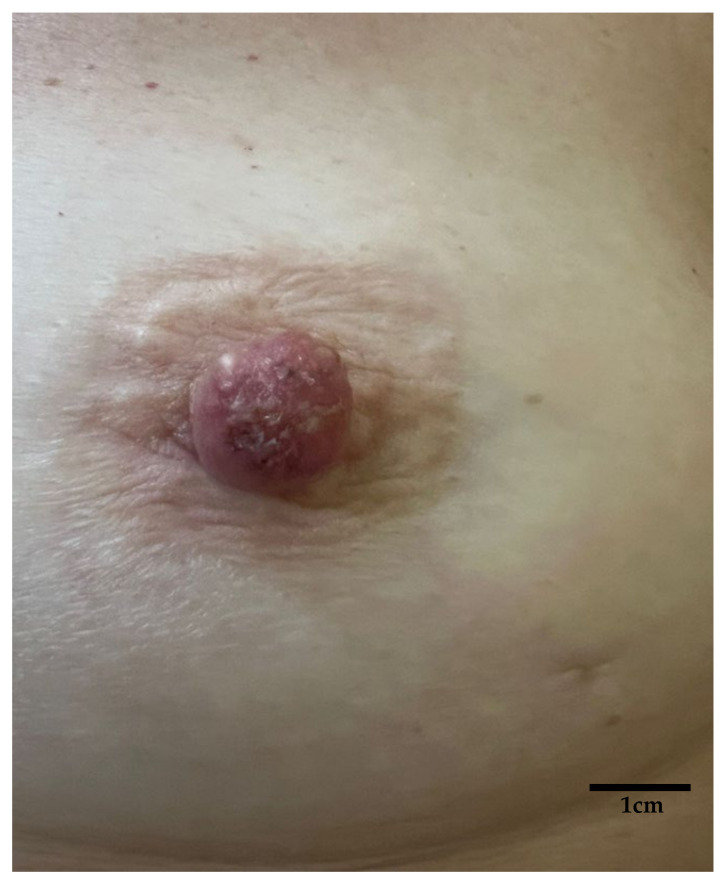
Macroscopic aspect: erythematous-crusty lesion of the left NAC with a 6-month history in a 56-year-old woman; no response to topical steroid ointment. Unilateral eczematous lesions of the NAC in postmenopausal women, especially if poorly responsive to topical treatment, should raise suspicion for malignant disease [1,2,3].

**Figure 2:**
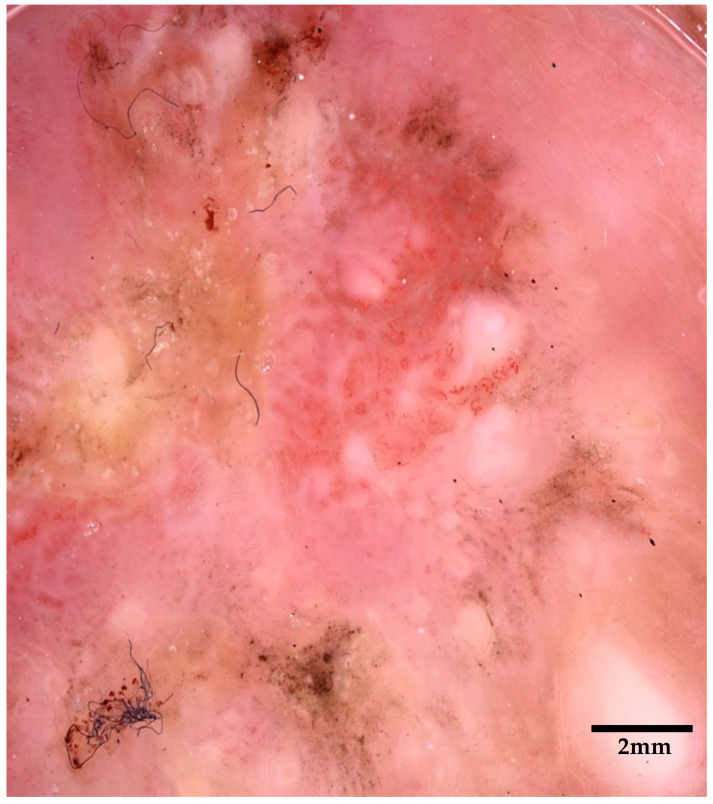
Dermoscopy showing a multicolor erythematous-yellowish lesion characterized by erosions, scales, dots, blotches, pseudocysts, and multicomponent vessel pattern: these features, though not specific, are more consistent with a suspect of a malignancy rather than inflammatory disease [4,5,6]. Sometimes pigmented variants, showing irregular brown pigmentation, gray dots, or globules, can mimic melanoma or pigmented basal cell carcinoma, complicating differential diagnosis [7,8].

**Figure 3:**
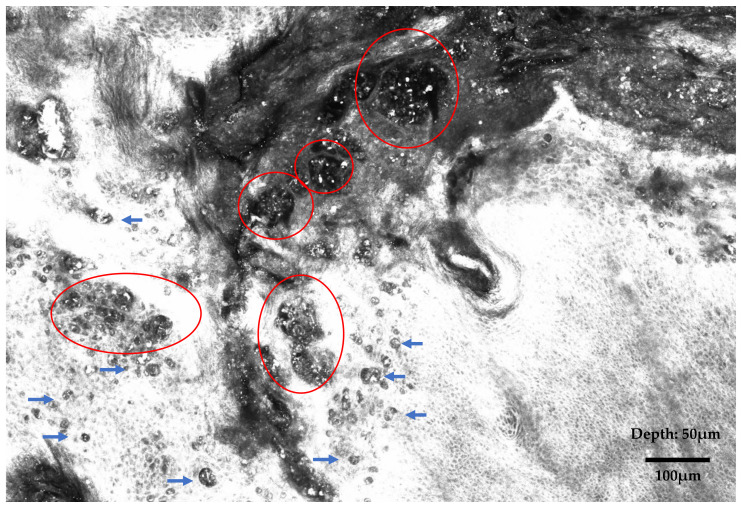
Reflectance confocal microscopy (RCM) is a non-invasive, in vivo imaging technique that enables real-time and high-resolution visualization of the epidermis and upper dermis at a near-histologic resolution: by employing a near-infrared laser and detecting backscattered light, RCM allows for horizontal optical sectioning of the skin, revealing cellular and architectural details without the need for biopsy [9,10,11]. In this case, normal epidermal honeycomb pattern is disrupted by the presence of big tumor nests (red circles), appearing as dark silhouettes of different sizes and shapes; these nests are composed of hyporeflective tumor cells (Paget cells) larger than adjacent keratinocytes with abundant, pale cytoplasm and small, mildly bright nuclei (blue arrowheads).

**Figure 4:**
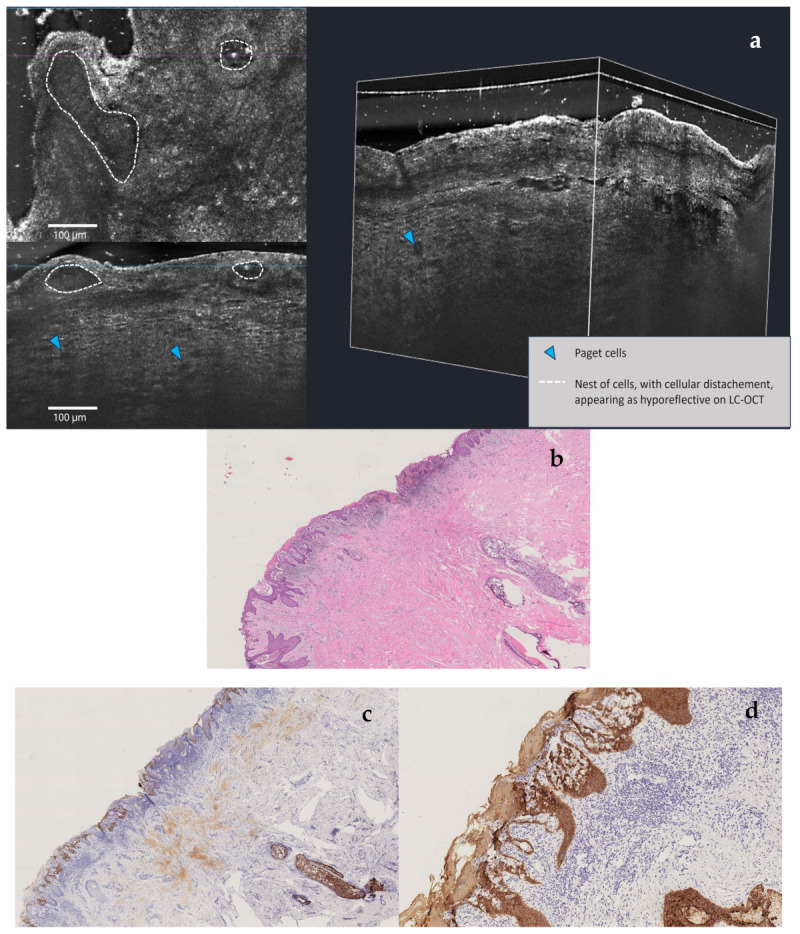
LC-OCT (**a**) is an innovative non-invasive imaging technique that uses light to capture high-resolution cross-sectional images of tissues [12]: in this patient, horizontal LC-OCT examination demonstrated the presence of a pseudovesicular pattern (white dotted lines) altering normal epidermal architecture, full of atypical cells (blue arrow) with mildly bright nuclei and several atypical cell nests. The vertical and 3D view captures the presence of abnormally large hyporeflective cells throughout the upper epidermis (Paget cells). After this non-invasive examination, it became evident that the patient was not affected by nipple eczema, which typically presents with intraepidermal vesicles (spongiotic pattern) filled with inflammatory cells and keratinocytes [13,14,15]. Therefore, after a multidisciplinary consultation, multiple nipple biopsies were carried out: a double cluster of R4a calcifications of the left breast was discovered. One cluster was based in the retro areolar region, with the other one in the lower outer quadrant (LOQ); one lymph node showing a slightly thickened cortex was also noted. Both clusters of microcalcification were removed by vacuum-assisted breast biopsy (VABB). Cytological examination of the nipple scraping and secretion was also performed. The nipple secretion was found to be inflammatory, and the nipple scraping showed isolated and loosely clustered malignant glandular cells with enlarged nuclei, prominent nucleoli, and pale cytoplasm, which is detected among squamous cells suspicious for Paget disease. A further VABB of the LOQ was operated and a moderately differentiated invasive breast carcinoma of no special type was ruled out. The lesion came back estrogen receptor (ER)-negative, progesterone receptor (PgR)-negative, and HER-2-positive, and Ki-67 was in the 25% range. Our breast multidisciplinary team chose a simple mastectomy as the treatment of choice: histological examination of this sample confirmed the presence of infiltrative carcinoma of the LOQ with adjacent ductal carcinoma in situ (DCIS) G2-G3 and the presence of DCIS G2-G3 of the large ducts behind the nipple and Paget disease of the nipple; a micrometastatic lymph node was diagnosed via molecular analysis and one-step nucleic acid analysis amplification (OSNA). The preliminary dermatological analysis of the NAC was very helpful in making the best therapeutic decision before confirming: these technologies provide high-resolution images of tissue architecture and cellular abnormalities, aiding in early detection and accelerating the need for biopsy [14,15]. However, clear data about the sensitivity and specificity of these non-invasive diagnostic techniques are still lacking, especially due to the low prevalence of Paget disease; in addition, in some microinvasive forms of Paget disease, RCM and LC-OCT examination could be negative. Therefore, histological confirmation remains essential: in our case, conventional instrumental investigations were negative, and cytology was nonspecific, so multiple targeted biopsies were required to confirm what had been immediately highlighted by high-resolution dermatological techniques. H&E stain low magnification (**b**): Paget disease of nipple and underlying DCIS of large milk ducts. CK7 stain low magnification (**c**): tumor cells are positive for CK7. CK5/6 stain low magnification (**d**): tumor cells are negative for CK5/6, and epidermis and skin adnexal structures are positive for CK5/6.

**Figure 5:**
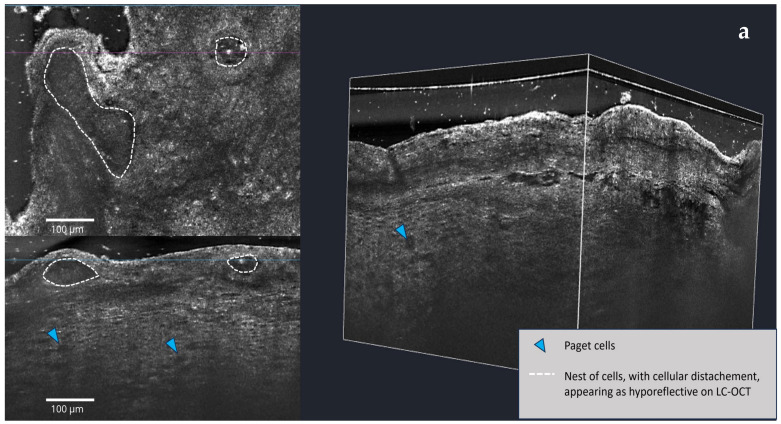

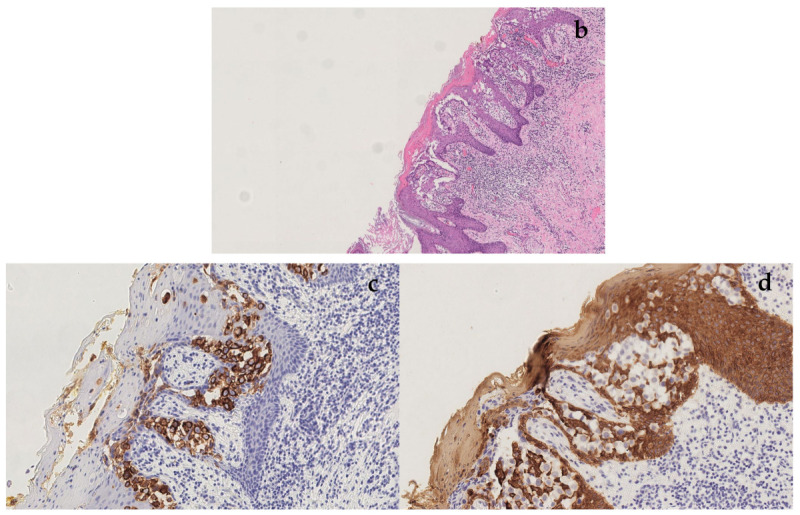
LC-OCT (**a**) examination. H&E stain high magnification (**b**): Paget disease of nipple and underlying DCIS of large milk ducts. CK7 stain high magnification (**c**): tumor cells are positive for CK7. CK5/6 stain high magnification (**d**): tumor cells are negative for CK5/6, and epidermis and skin adnexal structures positive for CK5/6.

## Data Availability

No new data were created or analyzed in this study.

## Abbreviations

The following abbreviations are used in this manuscript:

MPD	Mammary Paget disease
LC-OCT	Line-field confocal–optical coherence tomography
RCM	Reflectance confocal microscopy
NAC	Nipple areola complex
DCIS	Ductal carcinoma in situ
IDC	Invasive ductal carcinoma
LOQ	Lower outer quadrant
VABB	Vacuum-assisted breast biopsy
PgR	Progesterone Receptor
OSNA	One-step nucleic acid analysis amplification

## References

[1] Markarian S., Holmes D.R. (2022). Mammary Paget’s Disease: An Update. Cancers.

[2] Scott-Emuakpor R., Reza-Soltani S., Altaf S., NR K., Kołodziej F., Sil-Zavaleta S., Nalla M., Ullah M.N., Qureshi M.R., Ahmadi Y. (2024). Mammary Paget’s Disease Mimicking Benign and Malignant Dermatological Conditions: Clinical Challenges and Diagnostic Considerations. Cureus.

[3] Hudson-Phillips S., Cox K., Patel P., Al Sarakbi W. (2023). Paget’s disease of the breast: Diagnosis and management. Br. J. Hosp. Med..

[4] Apalla Z., Errichetti E., Kyrgidis A., Stolz W., Puig S., Malvehy J., Zalaudek I., Moscarella E., Longo C., Blum A. (2019). Dermoscopic features of mammary Paget’s disease: A retrospective case-control study by the International Dermoscopy Society. J. Eur. Acad. Dermatol. Venereol..

[5] Errichetti E., Avellini C., Pegolo E., De Francesco V. (2017). Dermoscopy as a Supportive Instrument in the Early Recognition of Erosive Adenomatosis of the Nipple and Mammary Paget’s Disease. Ann. Dermatol..

[6] Crignis G.S.N.D., Abreu L.D., Buçard A.M., Barcaui C.B. (2013). Polarized dermoscopy of mammary Paget disease. Bras. Dermatol..

[7] D’Erme A.M., Iozzo R., Viacava P., De Luca F., Janowska A., Dini V., Romanelli M., Fidanzi C., Bagnoni G. (2021). Pigmentary Mammary Paget Disease: Clinical, dermoscopical and histological challenge. Dermatol. Rep..

[8] Yanagishita T., Tamada Y., Tanaka M., Kasugai C., Takahashi E., Matsumoto Y., Watanabe D. (2011). Pigmented mammary Paget disease mimicking melanoma on dermatoscopy. J. Am. Acad. Dermatol..

[9] Shahriari N., Grant-Kels J.M., Rabinovitz H., Oliviero M., Scope A. (2021). Reflectance confocal microscopy. J. Am. Acad. Dermatol..

[10] Hofmann-Wellenhof R., Wurm E.M., Ahlgrimm-Siess V., Richtig E., Koller S., Smolle J., Gerger A. (2009). Reflectance Confocal Microscopy—State-of-Art and Research Overview. Semin. Cutan. Med. Surg..

[11] Levine A., Markowitz O. (2018). Introduction to reflectance confocal microscopy and its use in clinical practice. JAAD Case Rep..

[12] Cinotti E., Brunetti T., Cartocci A., Tognetti L., Suppa M., Malvehy J., Perez-Anker J., Puig S., Perrot J.L., Rubegni P. (2023). Diagnostic Accuracy of Line-Field Confocal Optical Coherence Tomography for the Diagnosis of Skin Carcinomas. Diagnostics.

[13] Richtig E., Ahlgrimm-Siess V., Arzberger E., Hofmann-Wellenhof R. (2011). Noninvasive differentiation between mamillary eczema and Paget disease by in vivo reflectance confocal microscopy on the basis of two case reports. Br. J. Dermatol..

[14] Dubois A., Levecq O., Azimani H., Siret D., Barut A., Suppa M., Del Marmol V., Malvehy J., Cinotti E., Rubegni P. (2018). Line-field confocal optical coherence tomography for high-resolution noninvasive imaging of skin tumors. J. Biomed. Opt..

[15] Donelli C., Suppa M., Tognetti L., Perrot J.L., Calabrese L., Pérez-Anker J., Malvehy J., Rubegni P., Cinotti E. (2023). Line-Field Confocal Optical Coherence Tomography for the Diagnosis of Skin Carcinomas: Real-Life Data over Three Years. Curr. Oncol..

